# Efficacy of combined periodontal-restorative treatment versus periodontal treatment alone for gingival recession associated with non-carious cervical lesions: a systematic review and meta-analysis

**DOI:** 10.1186/s12903-026-09404-1

**Published:** 2026-07-27

**Authors:** Heber Isac Arbildo-Vega, Franz Tito Coronel-Zubiate, Fredy Hugo Cruzado-Oliva, Rubén Aguirre-Ipenza, Sara Antonieta Luján-Valencia, Joan Manuel Meza-Málaga, Eduardo Luján-Urviola, Carlos Alberto Farje-Gallardo

**Affiliations:** 1 Faculty of Dentistry, Dentistry School, Universidad San Martín de Porres, Chiclayo, Peru; 2 Faculty of Human Medicine, Human Medicine School, Universidad San Martín de Porres, Chiclayo, Peru; 3 Graduate School, Universidad Nacional Toribio Rodríguez de Mendoza de Amazonas, Chachapoyas, Peru; 4 Faculty of Health Sciences, Stomatology School, Universidad Nacional Toribio Rodríguez de Mendoza de Amazonas, Chachapoyas, 01001 Peru; 5 Faculty of Stomatology, Stomatology School, Universidad Nacional de Trujillo, Trujillo, Peru; 6 Faculty of Health Sciences, Universidad Continental, Lima, Peru; 7 Faculty of Medicine, Medicine School, Universidad Católica de Santa María, Arequipa, Peru; 8 Faculty of Dentistry, Dentistry School, Universidad Católica de Santa María, Arequipa, Peru; 9 Postgraduate School, Universidad Católica de Santa María, Arequipa, Peru; 10 Faculty of Dentistry, Universidad Andina Néstor Cáceres Velásquez, Juliaca, Peru

**Keywords:** Periodontal treatment, Gingival recession, Non-carious cervical lesions, Review

## Abstract

**Objective:**

Gingival recession (GR) is frequently associated with non-carious cervical lesions (NCCLs), necessitating a combined periodontal and restorative approach for optimal clinical management. This systematic review and meta-analysis compared integrated periodontal-restorative interventions with isolated periodontal treatment for managing GRs linked to NCCLs.

**Methods:**

A comprehensive search of electronic databases, including PubMed, Cochrane Library, SciELO, Scopus, Web of Science, Embase, OpenAIRE, ProQuest, and Google Scholar, was performed up to December 2025 to identify randomized clinical trials (RCTs) with ≥ 1-year follow-up. We analyzed studies comparing integrated periodontal-restorative interventions against isolated periodontal therapy for NCCL-associated GRs. Risk of bias was assessed using RoB 2.0, while evidence certainty was graded using the GRADE framework.

**Results:**

Out of 199 screened records, four RCTs were included. Pooled data showed no significant differences between the treatment groups regarding nine periodontal parameters, with the exception of a slight increase in probing depth in the combined therapy groups.

**Conclusion:**

No statistically significant differences were observed between combined periodontal-restorative treatment and periodontal treatment alone for most clinical, esthetic, and root coverage outcomes in the management of gingival recessions associated with non-carious cervical lesions. The combined approach was associated with a significant reduction in dentin sensitivity and a statistically significant increase in probing depth, although the clinical relevance of the latter finding appears limited because it remained values within healthy periodontal ranges. These findings should be interpreted cautiously due to the low to very low certainty of evidence and the limited number of available randomized clinical trials. Further well-designed multicenter studies are needed to confirm the long-term effects of these interventions.

**Supplementary Information:**

The online version contains supplementary material available at https://doi.org/10.1186/s12903-026-09404-1.

## Background

Gingival recession (GR) is defined as a prevalent periodontal defect characterized by the apical displacement of the gingival margin relative to the cemento-enamel junction (CEJ), exposing the root surface to the oral environment [1–4]. Recent meta-analyses estimate that this condition affects approximately 81% of the global population (recessions ≥ 1 mm), aligning with global reports that place its prevalence in more than two-thirds of individuals [5, 6]. Its etiology is complex and multifactorial, involving anatomical, inflammatory, and traumatic factors that interact dynamically and may compromise periodontal stability over time [1, 3, 7–9]. However, the relative contribution of these factors remains incompletely understood, particularly in patients presenting concomitant cervical hard tissue defects.

Beyond root exposure, these lesions generate significant aesthetic and functional challenges that impair quality of life, such as the risk of root caries and the misalignment of gingival margins [3, 8]. One of the most recurrent complaints is dentin hypersensitivity, induced by the exposure of dentinal tubules [4, 8]. Under this premise, clinical management requires a comprehensive approach, especially when recession coexists with non-carious cervical lesions (NCCLs), forming a “combined defect” present in at least 50% of reported cases [1, 4, 10].

These cervical lesions are characterized by the loss of enamel or dentin at the dental neck due to mechanisms of abrasion, erosion, and abfraction [1, 11]. The interaction between both pathological processes alters the original root anatomy and suggests shared etiologies, such as traumatic toothbrushing, which drive the simultaneous progression of both conditions [4]. Moreover, the altered root anatomy generated by NCCLs may compromise flap adaptation, affect the stability of root coverage procedures, and influence the biological attachment response over restored surfaces, thereby increasing the complexity and unpredictability of clinical management. Consequently, the coexistence of these defects complicates the therapeutic approach, as it requires a coordinated intervention addressing both the gingival soft tissue and the loss of dental hard tissue [10].

CAF combined with connective tissue graft (CAF + CTG) is currently considered one of the most predictable approaches for the treatment of isolated gingival recessions without cervical lesions [1, 3]. This surgical approach seeks to maximize the percentage of root coverage, gain keratinized tissue, and ensure the stability of long-term results [8]. Nevertheless, in the presence of thin biotypes or multiple recessions, connective tissue substitutes such as dermal matrices or collagen have been proposed to improve gingival thickness and the predictability of clinical success [3].

When a concomitant NCCL is identified, the integration of adhesive restorative materials emerges as a necessary alternative to restore the relationship between tissues. Some protocols suggest that the restoration should precede the surgical act, especially in deep lesions (Type B + or V classification), while in less severe defects, the flap alone might be sufficient [10]. Although several clinical studies have suggested that adhesive restorative procedures performed prior to periodontal surgery may not negatively affect root coverage outcomes, the available evidence remains inconsistent and methodologically limited, particularly due to small sample sizes, variability in restorative materials, and differences in follow-up duration [1]. Furthermore, uncertainty persists regarding the biological behavior of soft tissue attachment over restored root surfaces and the long-term stability of these combined interventions.

Despite these advances, the management of combined defects remains controversial due to uncertainty regarding the predictability and long-term stability of root coverage over restored surfaces [8]. Although some clinical trials have reported no statistically significant differences between isolated periodontal treatment and combined periodontal-restorative approaches, these findings should be interpreted cautiously given the limited number of available randomized trials, heterogeneity in restorative protocols, and variability in outcome assessment methods [12–14]. Likewise, controversy exists regarding the biological response; ionomer offers antimicrobial properties, while resins present aesthetic stability and favorable surface texture if optimal polishing conditions are maintained [1, 4].

Current evidence regarding the management of gingival recessions associated with NCCLs remains limited and methodologically heterogeneous. Previous systematic reviews and meta-analyses, particularly that of Rovai et al. [15], suggested that combined periodontal-restorative approaches may achieve clinical outcomes comparable to periodontal surgery alone. However, the available evidence is still based on a small number of randomized clinical trials with limited sample sizes, short- to medium-term follow-up periods, and substantial methodological variability. Furthermore, previous evidence syntheses did not comprehensively integrate contemporary methodological approaches such as RoB 2.0 assessment, GRADE certainty evaluation, sensitivity analyses, or a broader assessment of patient-centered and esthetic outcomes, including dentin hypersensitivity, combined defect coverage, and professional esthetic evaluation.

In addition, uncertainty persists regarding the long-term predictability of root coverage over restored surfaces, the potential influence of different restorative materials, and the clinical significance of combined periodontal-restorative interventions compared with isolated periodontal treatment. These unresolved methodological and clinical issues justify the need for an updated systematic review and meta-analysis using contemporary evidence synthesis standards.

Therefore, the aim of the present systematic review and meta-analysis was to compare the clinical, esthetic, and patient-centered outcomes of combined periodontal-restorative treatment versus periodontal treatment alone for gingival recessions associated with NCCLs in adult patients. This review sought to provide an updated and methodologically rigorous synthesis of the available evidence to support clinical decision-making in the management of these combined defects.

## Methods

### Protocol and registration

The present systematic review and meta-analysis was conducted and reported in accordance with the Preferred Reporting Items for Systematic Reviews and Meta-Analyses (PRISMA 2020) statement [16]. The study protocol was prospectively registered in the International Prospective Register of Systematic Reviews (PROSPERO) under registration number CRD420261278151 [17].

### Definitions of interventions

- Combined periodontal-restorative treatment: This intervention involves the use of a Coronally Advanced Flap (CAF) in conjunction with a CTG and a restorative material—such as composite resin or resin-modified glass ionomer—for the management of GRs associated with NCCLs.
- Composite Resins (CR): These consist of light-cured, resin-based dental materials utilized as restorations within the experimental groups of the included studies.
- Resin-modified glass ionomer (RMGIC): These represent hybrid dental materials that integrate the fluoride-releasing and chemical-bonding properties of traditional glass ionomer cements with light-cured resins, serving as restorative agents for the experimental cohorts in the selected studies.

### Focused question and PICO framework

The focused question was formulated using the PICO format (population, intervention, comparison and outcomes), as detailed below:

- Population: Healthy adult patients (over 18 years old) presenting with GRs associated with NCCLs.
- Intervention: Combined periodontal-restorative treatment using connective tissue graft (CTG) and restorative materials (CR and RMGIC).
- Comparison: Periodontal treatment alone using coronally advanced flap (CAF) combined with connective tissue graft (CTG) without restoration of the cervical lesion.
- Outcomes: GR, probing depth (PD), clinical attachment level (CAL), keratinized tissue thickness (KTT), keratinized tissue width (KTW), aesthetic evaluation, dentin sensitivity (DS), combined defect height (CDH), combined defect coverage (CDC), root coverage (RC) and complete root coverage (CRC).

Focused question (PICO): Is integrated periodontal-restorative treatment more effective than periodontal treatment alone for treating gingival recessions concomitant with non-carious cervical lesions in adult patients?

### Information sources and search strategy

A comprehensive systematic search was conducted in the following electronic databases: PubMed, Cochrane Library, Scopus, Web of Science, Embase, and SciELO. Gray literature sources included Google Scholar, OpenAIRE, and ProQuest. Searches were performed from database inception until December 2025 without language or publication date restrictions. In addition, manual searches of the reference lists of all included studies were conducted to identify potentially relevant articles.

The search strategy combined controlled vocabulary terms and free-text keywords related to gingival recession, non-carious cervical lesions, connective tissue grafts, restorative materials, composite resins, and glass ionomer cements. The complete search strategies for all databases are presented in Table 1.

**Table 1:** Search and selection of studies

Database	Search Strategy	Number of study
PubMed	(((“Gingival Recession“[MeSH Terms] OR “Gingival Recession*“[Title/Abstract] OR “Recession, Gingival“[Title/Abstract])) AND ((“Tooth Wear“[MeSH Terms] OR “Non-carious cervical lesion*“[Title/Abstract] OR “NCCL“[Title/Abstract] OR “Cervical abrasion“[Title/Abstract] OR “Cervical erosion“[Title/Abstract]))) AND ((“Connective Tissue/transplantation“[MeSH Terms] OR “Connective tissue graft“[Title/Abstract] OR “CTG“[Title/Abstract]) AND (“Dental Restoration, Permanent“[MeSH Terms] OR “Composite Resins“[MeSH Terms] OR “Glass Ionomer Cements“[MeSH Terms] OR “Restorative material*“[Title/Abstract]))	17
Cochrane Library	( [mh “Gingival Recession”] OR “Gingival Recession*”:ti, ab, kw ) AND ( “Non-carious cervical lesion*”:ti, ab, kw OR “NCCL”:ti, ab, kw ) AND ( [mh ^"Connective Tissue/transplantation”] OR “Connective tissue graft”:ti, ab, kw ) AND ( “Restorative material*”:ti, ab, kw OR “Composite resin*”:ti, ab, kw )	1
Scopus	TITLE-ABS-KEY ( ( “Gingival Recession” OR “Recessão gengival” OR “Recesión gingival” ) AND ( “Non-carious cervical lesion*” OR “NCCL” OR “Cervical abrasion” ) AND ( “Connective tissue graft” OR “CTG” ) AND ( “Restorative material*” OR “Composite resin*” OR “Glass ionomer*” ) )	17
Web of Science	TS=((“Gingival Recession” OR “Recesión gingival”) AND (“Non-carious cervical lesion*” OR “NCCL” OR “Cervical abrasion”) AND (“Connective tissue graft” OR “CTG”) AND (“Restorative material*” OR “Composite resin*” OR “Glass ionomer*”))	100
Embase	(‘gingiva recession’/exp OR ‘gingiva recession’:ti, ab) AND (‘noncarious cervical lesion’/exp OR ‘non-carious cervical lesion*’:ti, ab OR ‘nccl’:ti, ab) AND (‘connective tissue graft’/exp OR ‘ctg’:ti, ab) AND (‘restorative material’/exp OR ‘composite resin’:ti, ab OR ‘glass ionomer’:ti, ab)	11
Scielo	(ti: (recesión gingival OR “gingival recession” OR “recessão gengival”)) AND (ab: (NCCL OR “lesión cervical no cariosa” OR “cervical lesion” OR “restorative”))	1
Google Scholar	(“Gingival recession”) AND (“Non-carious cervical lesion*” OR NCCL) AND (“Connective tissue graft” OR CTG) AND (“Restorative material*” OR “Composite resin*”) NOT (“Review” OR “In vitro”)	50
OpenAIRE	TITLE-ABS-KEY ( ( “Gingival Recession” OR “Recessão gengival” OR “Recesión gingival” ) AND ( “Non-carious cervical lesion*” OR “NCCL” OR “Cervical abrasion” ) AND ( “Connective tissue graft” OR “CTG” ) AND ( “Restorative material*” OR “Composite resin*” OR “Glass ionomer*” ) )	0
Proquest	(“Gingival recession”) AND (“Non-carious cervical lesion*” OR NCCL) AND (“Connective tissue graft” OR CTG) AND (“Restorative material*” OR “Composite resin*”) NOT (“Review” OR “In vitro”)	2

### Study selection

The study selection process was independently performed by two reviewers (HIA-V and FC-O). Studies were considered eligible if they met the following inclusion criteria: (1) randomized clinical trials (RCTs) with either parallel or split-mouth design; (2) adult patients (≥ 18 years) presenting gingival recessions associated with non-carious cervical lesions (NCCLs); (3) studies comparing combined periodontal-restorative treatment using coronally advanced flap (CAF) and connective tissue graft (CTG) associated with restorative materials versus periodontal treatment alone; (4) minimum follow-up period of 1 year; and (5) reporting at least one relevant clinical, esthetic, or patient-centered outcome related to root coverage procedures.

No publication year or language restrictions were applied. The exclusion criteria included: (1) non-randomized studies, case reports, case series, narrative reviews, systematic reviews, in vitro studies, animal studies, conference abstracts, and unpublished studies; (2) studies evaluating gingival recessions without associated NCCLs; (3) studies lacking a comparison group treated exclusively with periodontal surgery; and (4) duplicate or related publications reporting data from the same study population without providing additional relevant outcomes or follow-up information.

When multiple publications from the same clinical trial cohort were identified, all reports were carefully evaluated to determine whether they provided complementary outcomes or distinct follow-up assessments. In such cases, overlapping populations were considered during data extraction and synthesis to minimize the risk of participant double counting.

### Data extraction

A standardized and predefined data extraction form was developed for the present review. Two reviewers (FTC-Z and RA-I) independently extracted data from all eligible studies, including study characteristics, participant demographics, intervention details, follow-up duration, and clinical, esthetic, and patient-centered outcomes. Any disagreements were resolved through discussion and consensus with a third reviewer (SL-V).

### Risk of bias (RoB) assessment

The risk of bias of the included randomized clinical trials was independently assessed by two calibrated reviewers (FC-O and JM-M) using the Cochrane Risk of Bias 2.0 (RoB 2.0) tool [18]. Inter-reviewer agreement was high (k = 0.95), and disagreements were resolved through discussion with a third reviewer (HIA-V). The RoB 2.0 tool evaluates five domains: bias arising from the randomization process, deviations from intended interventions, missing outcome data, outcome measurement, and selection of the reported result. Each study was subsequently classified as presenting low risk of bias, some concerns, or high risk of bias.

### Statistical analysis and certainty of evidence (GRADE)

Statistical analyzes were performed using Review Manager software (RevMan version 5.3; Cochrane Collaboration, Oxford, UK). Continuous outcomes were pooled using the inverse variance method and expressed as mean differences (MDs) with 95% confidence intervals (CIs), whereas dichotomous outcomes were synthesized using the Mantel–Haenszel method and reported as odds ratios (ORs) with 95% CIs. Given the expected clinical and methodological heterogeneity among the included studies, a random-effects model was applied for all meta-analyses.

Statistical heterogeneity was assessed using Cochran’s Q test and quantified with the I² statistic. Heterogeneity was interpreted according to conventional thresholds, with I² values of 25%, 50%, and 75% representing low, moderate, and high heterogeneity, respectively. Statistical significance was set at *p* < 0.05.

The certainty of evidence for each outcome was assessed using the Grading of Recommendations Assessment, Development and Evaluation (GRADE) approach through the GRADEpro Guideline Development Tool (McMaster University and Evidence Prime Inc., Hamilton, Canada). The certainty of evidence was classified as high, moderate, low, or very low based on study limitations, inconsistency, indirectness, imprecision, and publication bias.

Sensitivity analyzes were performed using a leave-one-out approach to evaluate the robustness of the pooled estimates. Formal assessment of publication bias was not performed because fewer than ten studies were available for each outcome, consistent with current methodological recommendations for meta-analyses.

## Results

### Selection of studies

**Fig. 1:**
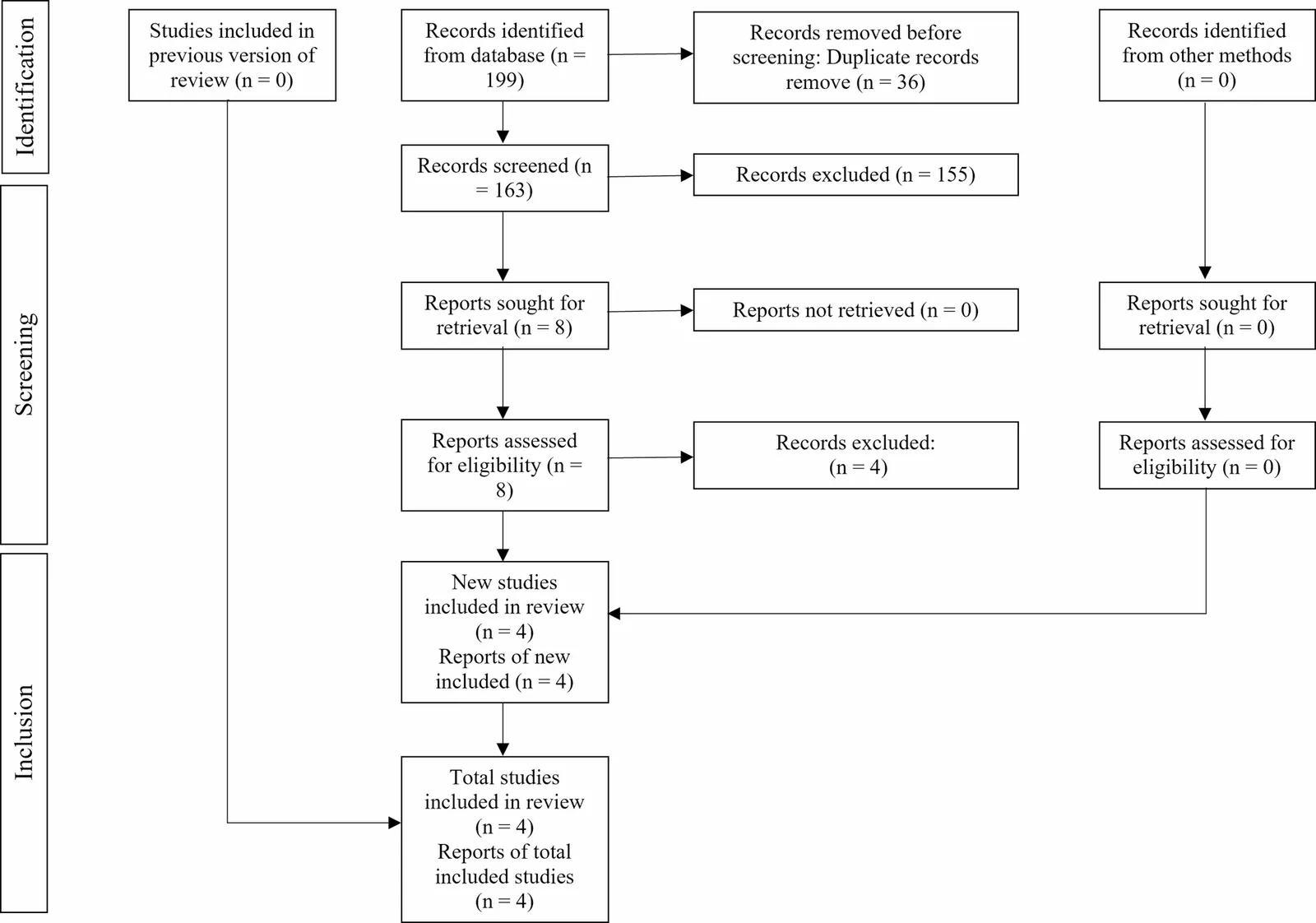
PRISMA flow diagram showing the selection process of studies included in the systematic review, from initial identification to final inclusion

The electronic and manual searches identified a total of 199 records. After the removal of 36 duplicates, 163 records remained for title and abstract screening. Of these, 155 studies were excluded because they did not meet the eligibility criteria. Consequently, 8 full-text articles were assessed for eligibility. After full-text evaluation, 4 studies were excluded for specific reasons detailed in Table 2, and 4 randomized clinical trials (RCTs) [19–22] were ultimately included in the qualitative and quantitative synthesis (meta-analysis) (Fig. 1).

**Table 2:** Reason for exclusion of studies

Author	Reason of exclusion
Santamaria et al. [23]	Did not include connective tissue graft (CTG) in the intervention protocol
de Sanctis et al. [24]	Lack of comparison group treated exclusively with periodontal surgery
Santamaria et al. [25]	Related publication derived from a previously reported clinical trial cohort
Santamaria et al. [14]	Insufficient eligibility for independent quantitative synthesis

### Characteristics of included studies

A total of four parallel-design randomized clinical trials (RCTs) [19–22] were included in the qualitative and quantitative synthesis. All studies were conducted in Brazil by the same research group (Santamaria et al.), which may limit the geographic generalizability and external validity of the available evidence. Careful evaluation of the included publications was performed to identify potential overlap among study populations. Santamaria et al. (2013) [22] and Santamaria et al. (2014) [21] were identified as related publications derived from the same clinical trial cohort but reporting different outcomes and follow-up assessments. In contrast, Santamaria et al. (2016) [20] and Santamaria et al. (2018) [19] represented separate randomized clinical trials with distinct participant cohorts and study objectives.

Across the included studies, the total number of participants ranged from 36 to 40, while the number of treated teeth ranged from 18 to 20 per group. The mean age of participants ranged from 35.6 to 44.5 years, and three studies [19, 21, 22] reported an age range between 19 and 71 years. Follow-up periods ranged from 1 to 2 years. Regarding restorative materials, two studies [19, 20] evaluated composite resin (CR), whereas two studies [21, 22] evaluated resin-modified glass ionomer cement (RMGIC) (Table 3).

**Table 3:** Characteristics of included studies

Authors	Year	Study design	Country	Number of patients (male/female)	Average age (range)	Follow–up	Groups	Number of patients per group	Number of teeth per group	Tooth type
Santamaria et al. [19]	2018	RCT parallel	Brazil	40 (22/18)	44.5 ± 10.6 (22–60)	1 year	CAF + CTG + Rc	20	20	7 canines and 13 premolars
							CAF + CTG	20	20	7 canines and 13 premolars
Santamaria et al. [20]	2016	RCT parallel	Brazil	36 (19/17)	37.05	1 year	CAF + CTG + Rc	18	18	13 canines and 5 premolars
							CAF + CTG	18	18	13 canines and 5 premolars
Santamaria et al. [21]	2014	RCT parallel	Brazil	36 (19/17)	35.6 (19–71)	2 years	CAF + CTG + R	18	18	8 canines and 10 premolars
							CAF + CTG	18	18	7 canines and 11 premolars
Santamaria et al. [22]	2013	RCT parallel	Brazil	36 (19/17)	35.6 (19–71)	2 years	CAF + CTG + R	18	18	8 canines and 10 premolars
							CAF + CTG	18	18	7 canines and 11 premolars

Authors	Groups	GR (mm)	PD (mm)	CAL (mm)	KTT (mm)	KTW (mm)	VAS	MRES	DS	DS (*n*)	CDH	CDC (%)	RC (%)	CRC (*n*)
Santamaria et al. [19]	CAF + CTG + Rc	6 m: 6.3 ± 1.7 1 y: 6.2 ± 1.8	6 m: 2.5 ± 0.5 1 y: 2.6 ± 0.7	6 m: 8.8 ± 1 1 y: 8.8 ± 1.8	6 m: 2.1 ± 0.6 1 y: 2 ± 0.7	6 m: 4.1 ± 0.9 1 y: 4.2 ± 1.7	6 m: 9.1 ± 2.2 1 y: 9.1 ± 1	NR	3 m: 1 ± 2.2 6 m: 0.6 ± 1.8	3 m: 4/20 6 m: 2/20	1 y: 3.3 ± 1	1 y: 75.3 ± 22.7	1 y: 93 ± 26.1	1 y: 12/20
	CAF + CTG	6 m: 6.7 ± 1.3 1 y: 6.8 ± 1.9	6 m: 2.1 ± 0.6 1 y: 2 ± 0.5	6 m: 8.7 ± 1.4 1 y: 8.8 ± 2	6 m: 2 ± 0.6 1 y: 1.9 ± 0.6	6 m: 4.1 ± 0.8 1 y: 4.1 ± 1.1	6 m: 9 ± 2.3 1 y: 9.2 ± 1.1	NR	3 m: 1.4 ± 1.7 6 m: 1.3 ± 2	3 m: 9/20 6 m: 9/20	1 y: 3.3 ± 0.7	1 y: 74.6 ± 31.5	1 y: 92.2 ± 28.4	1 y: 14/20
Santamaria et al. [20]	CAF + CTG + Rc	6 m: 10.1 ± 1.29 1 y: 10.01 ± 1.3	6 m: 2.77 ± 0.42 1 y: 2.66 ± 0.48	6 m: 12.88 ± 1.36 1 y: 12.67 ± 1.3	6 m: 2 ± 0.3 1 y: 1.97 ± 0.26	6 m: 2.59 ± 0.76 1 y: 2.73 ± 0.75	6 m: 8.61 ± 1.37 1 y: 8.66 ± 1.13	6 m: 7.05 ± 2.14 1 y: 7.52 ± 2.27	NR	6 m: 1/18	1 y: 3.73 ± 0.5	1 y: 73.84 ± 19.2	NR	1 y: 17/18
	CAF + CTG	6 m: 9.31 ± 1.6 1 y: 9.42 ± 1.5	6 m: 2.1 ± 0.47 1 y: 2 ± 0.48	6 m: 11.4 ± 1.7 1 y: 11.42 ± 1.6	6 m: 1.87 ± 0.47 1 y: 1.81 ± 0.44	6 m: 2.88 ± 0.96 1 y: 3 ± 0.9	6 m: 8.35 ± 2.24 1 y: 8.29 ± 2.3	6 m: 6.5 ± 2.2 1 y: 7.44 ± 2.3	NR	6 m: 8/18	1 y: 3.39 ± 0.57	1 y: 82.16 ± 16.1	NR	1 y: 15/18
Santamaria et al. [21]	CAF + CTG + R	NR	NR	NR	NR	NR	NR	6 m: 5.32 ± 1.9 1 y: 4.93 ± 2.5 2 y: 5 ± 2.5	NR	NR	NR	NR	NR	NR
	CAF + CTG	NR	NR	NR	NR	NR	NR	6 m: 6.7 ± 2.5 1 y: 6.8 ± 2.3 2 y: 7.3 ± 2.1	NR	NR	NR	NR	NR	NR
Santamaria et al. [22]	CAF + CTG + R	6 m: 9.48 ± 0.82 1 y: 9.51 ± 0.88 2 y: 9.57 ± 0.81	6 m: 2.15 ± 0.67 1 y: 2.12 ± 0.56 2 y: 2.11 ± 0.78	6 m: 11.63 ± 1.08 1 y: 11.51 ± 1.15 2 y: 11.57 ± 1.12	6 m: 1.95 ± 0.42 1 y: 1.81 ± 0.5 2 y: 1.87 ± 0.72	6 m: 3.34 ± 0.91 1 y: 3.38 ± 1.46 2 y: 3.56 ± 1.46	NR	NR	NR	NR	NR	2 y: 71.95 ± 13.25	2 y: 93.29 ± 7.97	2 y: 16/18
	CAF + CTG	6 m: 9.17 ± 1.53 1 y: 9.15 ± 1.46 2 y: 9.12 ± 1.52	6 m: 2.1 ± 0.55 1 y: 2 ± 0.45 2 y: 2 ± 0.34	6 m: 11.27 ± 1.17 1 y: 11.15 ± 1.72 2 y: 11.27 ± 1.7	6 m: 1.93 ± 0.53 1 y: 1.9 ± 0.77 2 y: 1.82 ± 0.44	6 m: 3.05 ± 1.11 1 y: 3.17 ± 1.5 2 y: 3.2 ± 1	NR	NR	NR	NR	NR	2 y: 79.31 ± 18.51	2 y: 91.56 ± 11.74	2 y: 15/18

*RCT* Randomized clinical trial, *CAF* Coronally advanced flap, *CTG* Connective tissue graft, *Rc* Resin composite, *R* Resin-modified glass ionomer, *GR* gingival recession, *PD* probing depth, *CAL* clinical attachment level, *KTT* Keratinized tissue thickness, *KTW* Keratinized tissue width, *VAS* Visual analog scale, *MRES* Modified root coverage esthetic score, *DS* dentin sensitivity, *CDH* Combined defect height, *CDC* Combined defect coverage, *RC* Root coverage, *CRC* complete root coverage, *NR* Not reported, *m* months, *y* years

Three studies [19, 20, 22] assessed gingival recession (GR), probing depth (PD), clinical attachment level (CAL), keratinized tissue thickness (KTT), keratinized tissue width (KTW), combined defect coverage (CDC), and complete root coverage (CRC). Esthetic outcomes were evaluated in three studies [19–21], whereas dentin sensitivity (DS) and combined defect height (CDH) were evaluated in two studies [19, 20]. Root coverage (RC) was evaluated in two studies [19, 22] (Table 3).

### Risk of bias analysis of studies

**Fig. 2:**
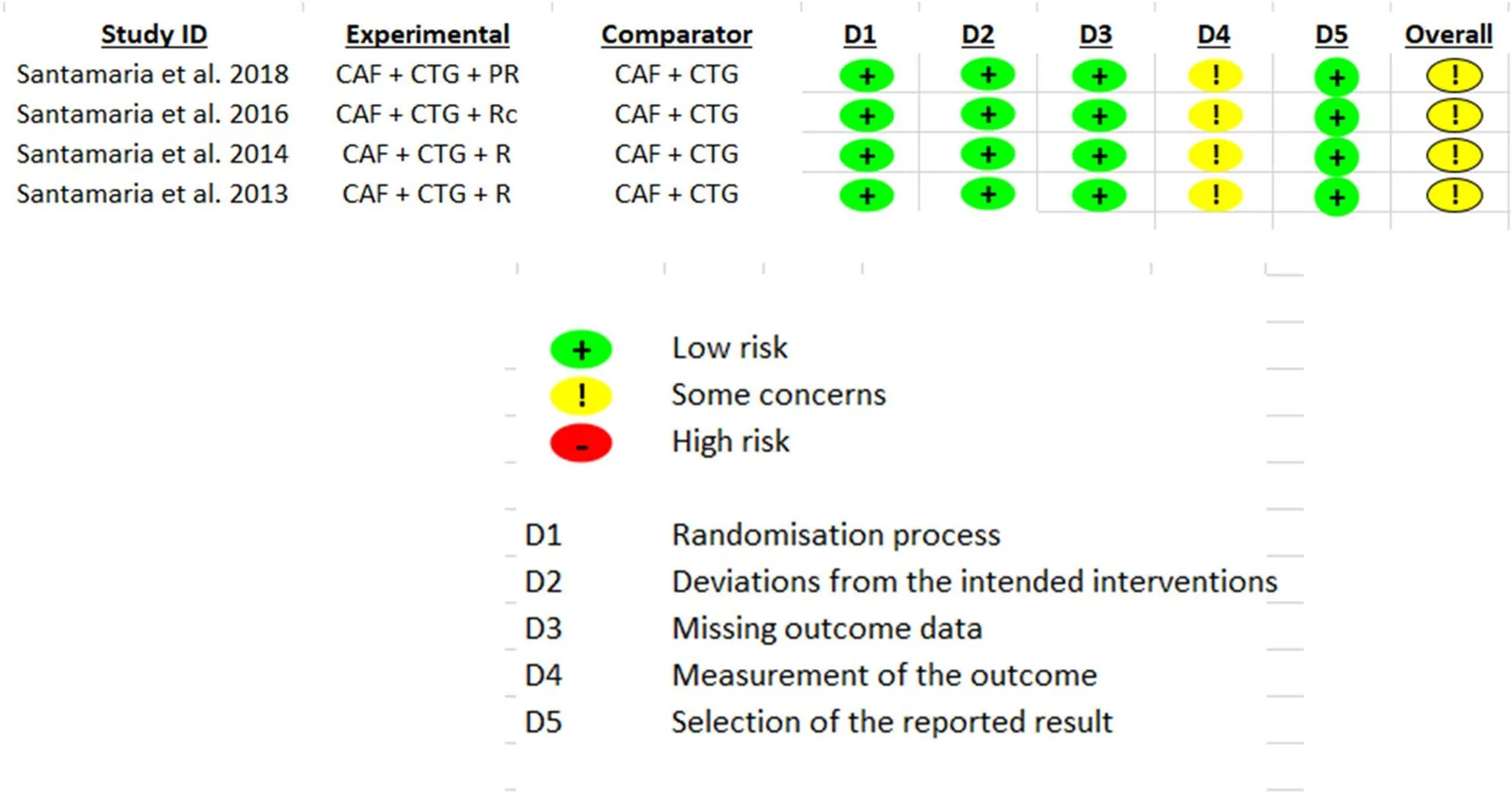
Risk of bias analysis of studies

All studies had a ‘some concerns’ risk of bias (Fig. 2).

### Synthesis of results (meta-analysis)

Three studies [19, 20, 22] evaluated the efficacy of combined periodontal-restorative treatment versus periodontal treatment alone for GRs associated with NCCLs. The parameters assessed included GR reduction, PD, CAL, KTT, KTW, CDC, and CRC. No statistically significant differences were observed across most clinical parameters, with the exception of PD, which was significantly higher in the combined treatment group in the combined treatment group. Esthetic outcomes were also evaluated in three studies [19–21], using the Visual Analog Scale (VAS) for patient assessment and the Modified Root Coverage Esthetic Score (MRES) for professional evaluation. Furthermore, CDH and RC were analyzed in two studies [19, 20]; neither these nor the esthetic parameters showed statistically significant differences. Finally, DS was assessed in two studies [19, 20], revealing a statistically significant difference for this specific parameter (Supplementary Material 1).

The pooled effect sizes were as follows: GR (MD = 0.24; 95% CI: -0.41–0.89; I² = 30.42%; *p* = 0.47); PD (MD = 0.47; 95% CI: 0.14–0.8; I² = 60.36%; *p* = 0.01); CAL (MD = 0.56; 95% CI: -0.18–1,3; I² = 36.93%; *p* = 0.14); KTT (MD = 0.13; 95% CI: -0,07–0.32; I² = 0.0%; *p* = 0.2); and KTW (MD = -0.04; 95% CI: -0.44–0.36; I² = 0.0%; *p* = 0.84). For esthetic outcomes, no significant differences were found in patient evaluation (MD = 0.01; 95% CI: -0.56–0.58; I² = 0.0%; *p* = 0.97) or professional evaluation (MD = -1.11; 95% CI: -3.44–1.22; I² = 79.3%; *p* = 0.35). Other parameters included DS (OR = 9.25; 95% CI: 2.39–35.77; I² = 0.0%; *p* = 0.00); CDH (MD = 0.23; 95% CI: -0.08–0.54; I² = 7.92%; *p* = 0.14); CDC (MD = -6.32; 95% CI: -13.4–0.75; I² = 0.0%; *p* = 0.08); RC (MD = 1.61; 95% CI: -4.5–7.72; I² = 0.0%; *p* = 0.61); and CRC (OR = 1.09; 95% CI: 0.41–2.91; I² = 0.0%; *p* = 0.87) (Supplementary Material 1).

### Sensitivity and publication bias analysis

Sensitivity analyzes were conducted using a leave-one-out approach to evaluate the robustness of the pooled estimates. Most outcomes remained relatively stable following the sequential removal of individual studies. However, greater variability was observed for probing depth (PD), professional esthetic assessment (MRES), and dentin sensitivity (DS), indicating that these outcomes were more sensitive to the influence of individual studies.

Formal assessment of publication bias was not performed because fewer than ten studies were available for each outcome, which limits the reliability and interpretability of funnel plots and regression-based methods for detecting small-study effects.

### GRADE analysis

The certainty of evidence was graded as very low for professional aesthetic assessments and low for the remaining clinical and aesthetic outcomes (Table 4). Consequently, further research is highly likely to have a substantial impact on the confidence of these effect estimates and is expected to change the current findings.

**Table 4:** GRADE analysis of included studies

Certainty assessment							*N*° of patients		Effect		Certainty
*N*° of studies	Study design	Risk of bias	Inconsistency	Indirectness	Imprecision	Other considerations	Combined periodontal- restorative treatment	Periodontal treatment only	Relative (95% CI)	Absolute (95% CI)	
Gingival recession (follow-up: range 1 year to 2 years)											
3	randomised trials	serious	not serious	not serious	serious	none	56	56	-	MD 0.24 mm higher (0.41 lower to 0.89 higher)	⨁⨁◯◯ Low
Probing depth (follow-up: range 1 year to 2 years)											
3	randomised trials	serious	serious	not serious	not serious	none	56	56	-	MD 0.47 mm higher (0.14 higher to 0.8 higher)	⨁⨁◯◯ Low
Clinical attachment level (follow-up: range 1 year to 2 years)											
3	randomised trials	serious	not serious	not serious	serious	none	56	56	-	MD 0.56 mm higher (0.18 lower to 1.3 higher)	⨁⨁◯◯ Low
Keratinized tissue thickness (follow-up: range 1 year to 2 years)											
3	randomised trials	serious	not serious	not serious	serious	none	56	56	-	MD 0.13 mm higher (0.07 lower to 0.32 higher)	⨁⨁◯◯ Low
Keratinized tissue width (follow-up: range 1 year to 2 years)											
3	randomised trials	serious	not serious	not serious	serious	none	56	56	-	MD 0.04 mm lower (0.44 lower to 0.36 higher)	⨁⨁◯◯ Low
Aesthetic evaluation of the patient (follow-up:1 year)											
2	randomised trials	serious	not serious	not serious	serious	none	38	38	-	MD 0.01 higher (0.56 lower to 0.58 higher)	⨁⨁◯◯ Low
Aesthetic evaluation of the professional (follow-up: range 1 year to 2 years)											
2	randomised trials	serious	very serious	not serious	serious	none	36	36	-	MD 1.11 lower (3.44 lower to 1.22 higher)	⨁◯◯◯ Very low
Dentin sensitivity (follow-up: 1 year)											
2	randomised trials	serious	not serious	not serious	serious	none	35/38 (92.1%)	21/38 (55.3%)	OR 9.25 (2.39 to 35.77)	367 more per 1,000 (from 194 more to 425 more)	⨁⨁◯◯ Low
Combined defect height (follow-up: 1 year)											
2	randomised trials	serious	not serious	not serious	serious	none	38	38	-	MD 0.23 mm higher (0.08 lower to 0.54 higher)	⨁⨁◯◯ Low
Combined defect coverage (follow-up: range 1 year to 2 years)											
3	randomised trials	serious	not serious	not serious	serious	none	56	56	-	MD 6.32% lower (13.4 lower to 0.75 higher)	⨁⨁◯◯ Low
Root coverage (follow-up: range 1 year to 2 years)											
2	randomised trials	serious	not serious	not serious	serious	none	38	38	-	MD 1.61% higher (4.5 lower to 7.72 higher)	⨁⨁◯◯ Low
Complete root coverage (follow-up: range 1 year to 2 years)											
3	randomised trials	serious	not serious	not serious	serious	none	45/56 (80.4%)	44/56 (78.6%)	OR 1.09 (0.41 to 2.91)	14 more per 1,000 (from 185 fewer to 129 more)	⨁⨁◯◯ Low

*CI*  confidence interval, *MD*  mean difference, *OR*  odds ratio

## Discussion

Our findings did not demonstrate statistically significant differences between combined periodontal-restorative treatment and periodontal treatment alone for most evaluated clinical and esthetic outcomes. However, these findings should be interpreted cautiously given the limited number of available randomized clinical trials and the low to very low certainty of evidence according to GRADE.

These findings are generally consistent with the previous systematic review and meta-analysis by Rovai et al. [15], which reported that prior restoration of NCCLs did not significantly influence root coverage predictability or CAL gain. However, the present review expands upon previous evidence syntheses by incorporating updated literature, formal RoB 2.0 assessment, GRADE certainty evaluation, sensitivity analyses, and a broader assessment of patient-centered and esthetic outcomes, including dentin hypersensitivity, combined defect coverage, complete root coverage, and professional esthetic evaluations. Despite these methodological advances, the available evidence remains limited and heavily dependent on a small number of trials conducted by the same research group. Similarly, Agossa et al. [4] reported comparable aesthetic and coverage results, although they highlighted a superior reduction in dentin hypersensitivity when combining restoration with a CTG. Our data showed no statistically significant differences in CRC or esthetic outcomes (VAS/MRES). Although a significant reduction in dentin sensitivity was observed in the combined treatment group, probing depth values were significantly higher in this group than in the periodontal treatment alone group. In this regard, Santamaría et al. [20] found drastic reductions in DS in the combined group (from 94% to 5.5%) compared to the surgery-only group (from 94% to 44.4%), suggesting that adhesive restoration provides added value in clinical comfort without compromising the biological response.

The high heterogeneity observed in the professional aesthetic evaluation (MRES) is likely driven by the type of restorative material employed. Our analysis of the included trials indicates that RMGI exhibits significant limitations in long-term color stability, with up to 44.1% of restorations failing to match tooth color after two years, thereby lowering aesthetic scores [21]. Conversely, nanofilled resin composites demonstrate superior color integration and stability, yielding higher and more predictable MRES scores [20]. These findings suggest that the choice of restorative material is a critical determinant of the long-term aesthetic success in combined periodontal-restorative procedures.

Recent studies have not demonstrated consistent differences between isolated periodontal treatment and combined periodontal-restorative approaches, although the certainty of the available evidence remains limited. Dursun et al. [26] compared isolated CTG against the use of various cervical filling materials (RMGI or giomer), finding no differences in periodontal measurements and achieving similar coverage across all groups (approx. 89–96%). Likewise, Isler et al. [27] demonstrated that the use of materials such as nanofilled resin or RMGI under CAFs does not alter parameters like PD or keratinized gingiva dimensions, although they noted a trend toward lower coverage with the use of giomer, underscoring the importance of material selection. Overall, the evidence suggests that, under an appropriate technique, the integration of restoration does not result in clinical detriment.

Regarding PD, our meta-analysis revealed that combined treatment leads to a statistically significant increase in PD values compared to periodontal treatment alone. This finding, although within healthy clinical limits (< 3 mm), is biologically explained by the formation of a longer junctional epithelium over the restorative material surface, which prevents the development of a new connective tissue attachment in the apical zone. Clinicians should consider that while the combined approach addresses the NCCL, it inherently modifies the gingival sulcus depth due to the subgingival presence of the restoration. Nevertheless, the observed increase remained within clinically healthy limits and should not be interpreted as evidence of periodontal deterioration.

Authors such as Pulido et al. [28] emphasize that correcting the NCCL improves post-surgical gingival contour and DS. However, they also point out that CTG treatment is intrinsically effective for improving the gingival phenotype, which may partially explain the absence of statistically significant differences observed across several outcomes in the present review. In contrast, Xie et al. [29] suggested that GR depth (GRD) influences treatment success more than cervical lesion depth (CLD), proposing that prior restoration might not be strictly necessary in all cases.

From a biological perspective, the efficacy of the combined approach is supported by the biocompatibility of modern materials. RMGI promote fibroblast adhesion and exhibit high retention and fluoride release [30]. For their part, composite resins allow for superior polishing, optimizing the emergence profile and reducing plaque accumulation [4]. Pathophysiologically, the marked reduction in DS in the combined group is due to the immediate sealing of dentinal tubules, eliminating external stimuli before gingival healing occurs.

This review presents significant limitations. The meta-analysis was based on four RCTs with small sample sizes (36–40 patients) and short-term follow-up (1–2 years). An additional limitation is that all included randomized clinical trials were conducted in Brazil by the same research group (Santamaria et al.), which may restrict the external validity and generalizability of the findings. Furthermore, some publications represented related reports derived from the same clinical trial populations, although careful assessment was performed to minimize the risk of participant double counting during data synthesis. The dependence of the available evidence on a single research group highlights the need for independent multicenter randomized clinical trials conducted in diverse populations and clinical settings. Furthermore, the certainty of evidence for most evaluated outcomes was rated as low or very low according to GRADE, mainly due to imprecision arising from small sample sizes, limited numbers of studies, and concerns regarding risk of bias. Therefore, confidence in the pooled estimates remains limited.

Although sensitivity analyzes showed relatively stable pooled estimates for most outcomes, these findings should be interpreted cautiously because the certainty of evidence was predominantly low or very low according to GRADE. Consequently, the true effect may differ substantially from the current estimates, and future well-designed randomized clinical trials are likely to influence both the magnitude and direction of the observed effects. Publication bias could not be reliably assessed because fewer than ten studies were available for each outcome. Consequently, confidence in the pooled estimates remains limited, and future well-designed randomized clinical trials are likely to modify the current understanding of the comparative effects of these interventions. The strength of this work lies in its rigorous methodology and the direct comparison of current evidence, providing a critical foundation for clinical decision-making.

The available evidence does not demonstrate a clear superiority of one approach over the other regarding root coverage outcomes. However, because the certainty of evidence was low to very low for most outcomes, these findings should not be interpreted as evidence of therapeutic equivalence. Rather, they indicate that current evidence is insufficient to establish whether meaningful differences exist between the interventions. Therefore, treatment decisions should be individualized according to patient characteristics, clinical presentation, and practitioner judgment [20]. In cases of shallow recessions or when a minimally invasive approach is preferred, surgery may be prioritized, with the restoration evaluated at a later stage. Future multicenter studies with longer follow-up periods are required to raise the level of evidence and confirm whether the benefits of the combined approach are maintained in the long term.

## Conclusions

Based on the available evidence from this systematic review and meta-analysis, no statistically significant differences were observed between combined periodontal-restorative treatment and periodontal treatment alone for most clinical, esthetic, and root coverage outcomes in the management of gingival recessions associated with non-carious cervical lesions over follow-up periods ranging from one to two years. The combined approach was associated with a significant reduction in dentin sensitivity and a statistically significant increase in probing depth; However, the clinical relevance of the latter finding appears limited because the reported values remained within healthy periodontal ranges.

These findings should be interpreted with caution because the certainty of evidence was rated as low to very low according to GRADE, all included studies presented some concerns regarding risk of bias, and the available evidence was derived from a small number of trials conducted by a single research group. Therefore, the absence of statistically significant differences should not be interpreted as evidence of therapeutic equivalence between the interventions. Rather, current evidence remains insufficient to establish whether meaningful differences exist between the two approaches. Consequently, confidence in the current estimates is limited, and future well-designed multicenter randomized clinical trials with longer follow-up periods are likely to influence the magnitude and direction of the observed effects.

## Supplementary Information


Supplementary Material 1: Forest plots and leave-one-out sensitivity analyses for outcomes included in the meta-analysis. Figure S1. GR between combined periodontal-restorative treatment and periodontal treatment alone for GRs associated with NCCLs. Figure S2. PD between combined periodontal-restorative treatment and periodontal treatment alone for GRs associated with NCCLs. Figure S3. CAL between combined periodontal-restorative treatment and periodontal treatment alone for GRs associated with NCCLs. Figure S4. KTT between combined periodontal-restorative treatment and periodontal treatment alone for GRs associated with NCCLs. Figure S5. KTW between combined periodontal-restorative treatment and periodontal treatment alone for GRs associated with NCCLs. Figure S6. Aesthetic evaluation for the patients between combined periodontal-restorative treatment and periodontal treatment alone for GRs associated with NCCLs. Figure S7. Aesthetic evaluation for the professional between combined periodontal-restorative treatment and periodontal treatment alone for GRs associated with NCCLs. Figure S8. DS between combined periodontal-restorative treatment and periodontal treatment alone for GRs associated with NCCLs. Figure S9. CDH between combined periodontal-restorative treatment and periodontal treatment alone for GRs associated with NCCLs. Figure S10. CDC between combined periodontal-restorative treatment and periodontal treatment alone for GRs associated with NCCLs. Figure S11. RC between combined periodontal-restorative treatment and periodontal treatment alone for GRs associated with NCCLs. Figure S12. CRC between combined periodontal-restorative treatment and periodontal treatment alone for GRs associated with NCCLs.


## Data Availability

The dataset supporting the conclusion of this article is included within the article. However, additional information can be requested from the corresponding author upon reasonable inquiry.

## Abbreviations

GR: Gingival Recession
NCCL: Non-Carious Cervical Lesions
RCTs: Randomized Clinical Trials
CEJ: Cemento-enamel junction
CAF: Coronally Advanced Flap
CTG: Connective Tissue Graft
RMGI: Resin-Modified Glass Ionomer
GRADE: Grading of Recommendations Assessment, Development and Evaluation
PRISMA: Preferred Reporting Items for Systematic Reviews and Meta-Analyses
PROSPERO: International Prospective Register of Systematic Reviews
CR: Composite Resins
PICO/PECO: Population, Exposure, Comparator, Outcome
PD: Probing Depth
CAL: Clinical Attachment Level
KTT: Keratinized Tissue Thickness
KTW: Keratinized Tissue Width
DS: Dentin Sensitivity
CDH: Combined Defect Height
CDC: Combined Defect Coverage
RC: Root Coverage
CRC: Complete Root Coverage
VAS: Visual Analog Scale
MRES: Modified Root Coverage Esthetic Score
HR: Hazard Ratio
CI: Confidence Interval
OR: Odds Ratio
