# Supplementary material for: Efficacy of combined periodontal-restorative treatment versus periodontal treatment alone for gingival recession associated with non-carious cervical lesions: a systematic review and meta-analysis
Source: BMC Oral Health. 2026 Jul 27;26:1639. doi: 10.1186/s12903-026-09404-1 (PMC13527942; doi:10.1186/s12903-026-09404-1)
Supplement: Supplementary file 1 [file 12903_2026_9404_MOESM1_ESM.docx]

**Figure 1.** GR between combined periodontal-restorative treatment and periodontal treatment alone for GRs associated with NCCLs

Eggers Test = 0.1463

**Figure 2.** PD between combined periodontal-restorative treatment and periodontal treatment alone for GRs associated with NCCLs

Eggers Test = 0.2796

**Figure 3.** CAL between combined periodontal-restorative treatment and periodontal treatment alone for GRs associated with NCCLs

Eggers Test = 0.4325

**Figure 4.** KTT between combined periodontal-restorative treatment and periodontal treatment alone for GRs associated with NCCLs

Eggers Test = 0.7693

**Figure 5.** KTW between combined periodontal-restorative treatment and periodontal treatment alone for GRs associated with NCCLs

Eggers Test = 0.2462

**Figure 6.** Aesthetic evaluation for the patients between combined periodontal-restorative treatment and periodontal treatment alone for GRs associated with NCCLs

Eggers Test = 0.4954

**Figure 7.** Aesthetic evaluation for the professional between combined periodontal-restorative treatment and periodontal treatment alone for GRs associated with NCCLs

Eggers Test = 0.3616

**Figure 8.** DS between combined periodontal-restorative treatment and periodontal treatment alone for GRs associated with NCCLs

Harbord Test = 0.7876

**Figure 9.** CDH between combined periodontal-restorative treatment and periodontal treatment alone for GRs associated with NCCLs

Eggers Test = 0.4864

**Figure 10.** CDC between combined periodontal-restorative treatment and periodontal treatment alone for GRs associated with NCCLs

Eggers Test = 0.3976

**Figure 11.** RC between combined periodontal-restorative treatment and periodontal treatment alone for GRs associated with NCCLs

Eggers Test = 0.9225

**Figure 12.** CRC between combined periodontal-restorative treatment and periodontal treatment alone for GRs associated with NCCLs

Harbord Test = 0.1998
